# Benidipine calcium channel blocker promotes the death of cigarette smoke-induced senescent cells and improves lung emphysema

**DOI:** 10.18632/aging.205259

**Published:** 2023-12-12

**Authors:** Alberta Palazzo, Gabriela Makulyte, Delphine Goerhig, Jean-Jacques Médard, Vincent Gros, François Trottein, Serge Adnot, David Vindrieux, Jean-Michel Flaman, David Bernard

**Affiliations:** 1 Centre de Recherche en Cancérologie de Lyon, Inserm U1052, CNRS UMR 5286, Centre Léon Bérard, Université de Lyon, Lyon, France; 2 Equipe Labellisée la Ligue Contre le Cancer, Lyon, France; 3 Université Paris Est Créteil, INSERM U955, IMRB, Créteil F-94010, France; 4 AP-HP, Hôpital Henri Mondor, Département de Physiologie-Explorations Fonctionnelles and FHU Senec, Créteil F-94010, France; 5 Université Lille, CNRS, INSERM, CHU Lille, Institut Pasteur de Lille, U1019 - UMR 9017 - CIIL - Center for Infection and Immunity of Lille, Lille F-59000, France

**Keywords:** senolytic, cigarette smoke, lung disease, cellular senescence, calcium channel

## Abstract

Smoking is the main risk factor for many lung diseases including chronic obstructive pulmonary disease. Cigarette smoke (CS) contains carcinogenic and reactive oxygen species that favor DNA mutations and perturb the homeostasis and environment of cells. CS induces lung cell senescence resulting in a stable proliferation arrest and a senescence-associated secretory phenotype. It was recently reported that senescent cell accumulation promotes several lung diseases. In this study, we performed a chemical screen, using an FDA-approved drug library, to identify compounds selectively promoting the death of CS-induced senescent lung cells. Aside from the well-known senolytic, ABT-263, we identified other potentially new senescence-eliminating compounds, including a new class of molecules, the dihydropyridine family of calcium voltage-gated channel (CaV) blockers. Among these blockers, Benidipine, decreased senescent lung cells and ameliorates lung emphysema in a mouse model. The dihydropyridine family of CaV blockers thus constitutes a new class of senolytics that could improve lung diseases. Hence, our work paves the way for further studies on the senolytic activity of CaV blockers in different senescence contexts and age-related diseases.

## INTRODUCTION

The lung is the first organ impacted by cigarette smoke (CS). Prolonged CS exposure accelerates lung alterations including inflammation, emphysema, fibrosis and chronic obstructive pulmonary disease (COPD) during aging, and is even thought to accelerate aging [1, 2].

A major effect of exposure to CS is the induction of cellular senescence [3–6]. This is characterized by a stable proliferation arrest and the acquisition of a specific senescence-associated secretory phenotype or SASP, containing pro-inflammatory cytokines, immune modulators as well as metalloproteases. Senescence is considered to favor numerous age-related diseases as depletion of senescent cells increases lifespan and healthy lifespan. Cellular senescence is proposed to favor aging and aging-related diseases likely by limiting the proliferative capacity of tissues, by inducing inflammation and/or by favoring disorganization of tissue architecture and fibrosis [7–10].

Senescent cell accumulation has also been associated with an increased risk of lung diseases, fostered by exposure to CS [7–10]. In the lung, decreasing cellular senescence or depleting senescent cells have been shown to improve lung alterations including fibrosis and emphysema [11–15], thus advocating for the use of senolytic compounds to kill lung senescent cells and improve pulmonary health. Here, our aim was to identify senolytic compounds in the context of CS-induced senescence and to assess whether they improved lung emphysema.

## RESULTS

**Figure 1:**
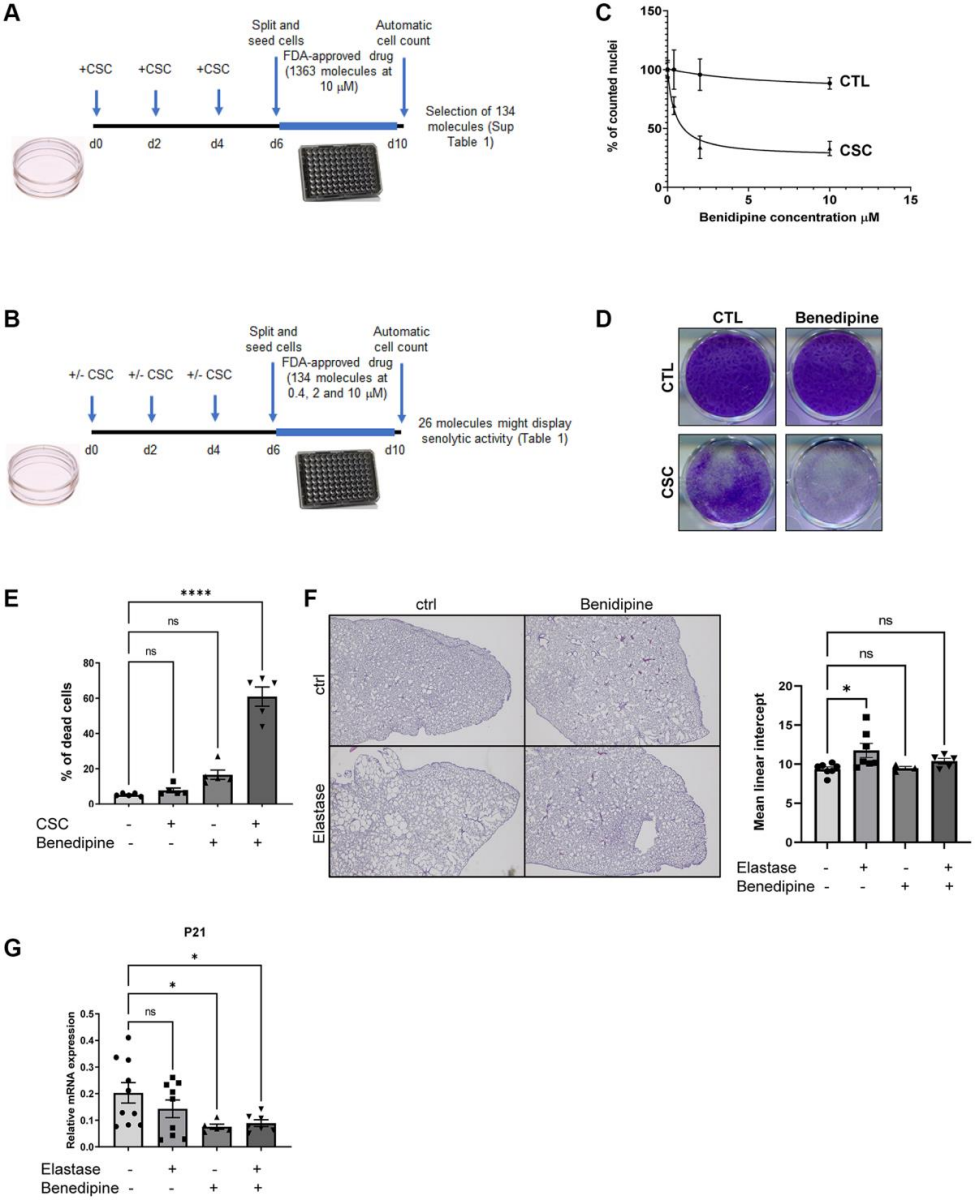
**Identification of CaV blockers as a potential new class of senolytics.** (**A**) Schematic representation of the experimental strategy used to identify molecules able to decrease the quantity of CSC-induced senescent cells. (**B**) Summary of the procedure used to validate which molecules among the 26 identified in (**A**) display senolytic activity on CSC-induced senescent cells. (**C**) Dose response curve of CaV blocker Benidipine senolytic activity. X axis shows Benedipine contrations used, y axis shows pourcentage of cell count relative to control DMSO treated cells (% of counted nuclei) (mean ± SEM). (**D**) After paraformaldehyde fixation, cells were crystal violet stained to visualize cell density. (**E**) Cells were collected, incubated with trypan blue before being counted. The percentage of dying cells (trypan blue-positive) is shown. (mean ± SEM, One-way ANOVA statistical test, *n* = 5). (**F**) Mice were treated once with elastase or vehicle and five time a week with Benidipine or vehicle. Three weeks after elastase injection, lungs were fixed, embedded and slides stained by hematoxylin/eosin. The left panel shows representative images of lungs for each experimental condition. The right panel shows quantification of emphysema by calculating the mean linear intercept. (mean ± SEM, One-way ANOVA statistical test, ctrl: *n* = 7; elastase: *n* = 7; Benidipine: *n* = 3; elastase + Benidipine: *n* = 5). (**G**) RT-qPCR results performed on RNA samples extracted from lungs of mice treated or not with Elastase and/or Benedipine. ^*^p < 0.05; ^****^p < 0.0001.

As expected, CS condensates (CSC) induced premature senescence of normal human lung fibroblasts (MRC5) as evidenced by: (i) proliferation arrest it provoked as CS decreased cell density (Supplementary Figure 1A), diminished level of KI67 proliferation marker (Supplementary Figure 1B), increased expression of cyclin-dependent kinase inhibitors CDKN1A/p21 (Supplementary Figure 1B) and molecular signatures associated with proliferation arrest (Supplementary Figure 1C); (ii) an increase in SA-β-Gal activity (Supplementary Figure 1D); (iii) an increase in the expression of SASP factors and associated molecular signatures (Supplementary Figure 1E–1G). These senescent cells were used for 2 sequential drug screens using an FDA-approved library, to identify compounds able to kill CS-induced senescent cells. In the first screen, 1,363 molecules were tested on senescent cells at 10 μM. We then discarded conventional chemotherapies and toxic molecules, which resulted in 134 molecules that strongly decreased the number of nuclei (Figure 1A and Supplementary Table 1). These molecules were then tested in control and CS-induced senescent cells at 3 different concentrations (Figure 1B), and among these, 26 potentially killed CS-induced senescent cells (Table 1).

**Table 1:** List of molecules that may display senolytic activity during cigarette smoke-induced senescence.

**Molecules**	**Targets**	**Senolytic activity proposed**
ABT-263	Anti-apoptotic BCL2, BCLxL, BCLW inhibitors	https://doi.org/10.1111/acel.12445 https://doi.org/10.1038/nm.4010
Aprotinin	Serine protease inhibitors	Not described (nd)
Belinostat	Histone deacetylase inhibitor	nd
Benidipine	Voltage-gated calcium channel (CaV) inhibitor	nd
Birinapant	Smac mimetic	https://doi.org/10.1101/2022.04.01.486768
Cilnipidine	CaV inhibitor	nd
Cinacalcet	allosteric agonist of Ca receptor	nd
Cyclosporine A	Calcineurin, mPTP opening	WO2018215795A2
Dabigatran etexilate mesylate	Thrombin inhibitor	nd
DHEA	Steroid hormone	nd
Domiphen bromide	Chemical antiseptic	
Ebastine	H1-histamine receptor antagonist	nd
Enzastaurin (LY317615)	Serine/threonine kinase inhibitor	WO2015116735A1
Erlotinib	EGFR inhibitor	nd
Erlotinib	EGFR inhibitor	nd
Felodipine	CaV inhibitor	nd
Flunarizine	Na+/Ca2+ channel (T-type) blocker	nd
Idebenone	Coenzyme Q10 analog, antioxidant	nd
Lacidipine	CaV inhibitor	nd
Oltipraz	NRF2 inhibitor	nd
Orlistat	Pancreatic and gastric lipases inhibitor, FASN inhibitor	nd
PCI-32765 (Ibrutinib)	BTK inhibitor	nd
Regorafenib	Multi-targeted receptor tyrosine kinase inhibitor	nd
Suprofen	COX-1 and -2 inhibitors	EP18202657A1
Trandolapril	ACE inhibitor	nd
Trifluoperazine	Dopamine D2 receptor inhibitor	nd

Experiments were performed using 134 selected molecules as described in Figure 1B. Here, the molecules that decreased the number of CS-induced senescent cells vs. vehicle treated CS-induced senescent cells with at least 2 different concentrations (0.4, 2 and 10 mM were tested for each molecule) are listed. Erlotinib was identified twice as it was in 2 different formulations in the initial library. The underlined molecules targets CaV channels.

Aside from the well-known ABT-263 senolytic compound [16, 17], our screens identified Birinapant, a Smac mimetic with a reported senolytic activity [18], and three other compounds proposed to exert senolytic activity in filled patents (Table 1). Strikingly, 4 different molecules targeting voltage-gated calcium channels (CaV), namely Benidipine, Cilnidipine, Felodipine and Lacidipine, displayed potential senolytic activities on CS-induced senescent cells. To confirm this senolytic activity, we assessed Benidipine, or Cilnidipine, on control and CS-induced senescent cells and observed a clear specific decrease cell number and density of senescent cells without impacting the non-senescent cells (Figure 1C, 1D and Supplementary Figure 2A, 2B). This decreased cell number correlated with senescent cell death as assessed by the quantity of blue trypan positive cells (Figure 1E).

Destruction of lung parenchyma and induction of lung emphysema upon exposure to CS is mediated by elastase activity. Intra-tracheal elastase injection is thus frequently used to better understand mechanisms regulating and impacting the development of lung emphysema [19–23]. During elastase treatment, elimination of senescent cells, by ABT-263 or by depleting p19^arf^-positive cells, decreases emphysema [14]. Here, we assessed whether Benidipine treatment exerted similar protective effects against elastase-induced emphysema. At necropsy, lung tissues were inflated with fixative under constant pressure to avoid altering lung morphology. As expected, elastase induced a significant change in lung morphology and in the alveolar mean linear intercept, which reflects the mean alveolar size (Figure 1F). Strikingly, these lung alterations were strongly reduced when mice were treated with Benidipine (Figure 1F). This was correlated with a decrease in senescent cells according to level of p21 senescence marker (Figure 1G). Together Benidipine mimicked the effect of eliminating senescent cells during elastase-induced emphysema [14] strongly supporting that Benidipine is acting as a senolytic. We did not observe increase expression of p21 senescence marker 3 weeks after elastase treatment, suggesting that either increase of senescent cells occurred earlier or/and the increase of senescent cells was masked by complex lung remodeling after elastase treatment. These results are in line with the ones observed by [14]. It will also be interesting to extend these observations in the future in additional model of lung emphysema such as during CS exposure or during aging.

Hence, Benidipine, a dihydropyridine CaV blocker used to lower blood pressure, could constitute a new senolytic compound targeting lung senescent cells, as it herein improved lung emphysema. We also identified other dihydropyridine CaV blockers that may cause CS-induced senescent cell death, some of which including Benidipine improve pulmonary fibrosis [24], a process also promoted by lung senescent cells [15]. This suggests that dihydropyridine family of CaV blockers could constitute a new class of senolytics that could improve lung diseases, in particular in the context of heavy smokers, a population with a higher risk of developing interconnected lung diseases, including fibrosis, COPD and cancers [25–28]. Collectively, this work offers novel perspectives for the use of CaV blockers to improve other age-related diseases that are promoted by senescent cells.

## MATERIALS AND METHODS

### Cell culture and reagents

MRC5 normal human fibroblasts (ATCC) were cultured in Dulbecco’s modified Eagle’s medium (DMEM, Life Technologies, USA) with GlutaMax and supplemented with 10% FBS (Sigma-Aldrich, USA) and 1% penicillin/streptomycin (ThermoFisher Scientific, USA). Cells were maintained at 37°C under a 5% CO2 atmosphere. All experiments were carried out on cells at early passages (between 22 and 28). A DiscoveryProbe FDA-approved Drug Library (Clinisciences, France), Benidipine (Clinisciences) and cigarette smoke condensates (Murty Pharmaceuticals, USA) were used at the concentrations indicated in the figures.

### RNA extraction, reverse transcription and real-time quantitative PCR

Total RNAs were extracted with phenol-chloroform using Upzol (Dutscher, Brumath, France). cDNAs were synthetized using the Maxima First cDNA Synthesis Kit (ThermoFisher Scientific). Quantitative PCR (qPCR) were performed by combining cDNA mixed with primers (200 nM), SYBR™ Green PCR Master Mix for mouse genes (ThermoFisher Scientific) or TaqMan mix for human genes (Roche, Switzerland) and Universal Probe Library probes (100 μM) (ThermoFisher Scientific) for the gene of interest. qPCR analyses were carried out with the CFX96 Thermocycler (Bio-Rad, USA). Relative mRNA levels were calculated using the Comparative Ct (ΔΔCT) method. Gene expression was normalized against GAPDH. Primer sequences used are listed in Supplementary Table 2.

### Senescence-associated β-Galactosidase analysis and Crystal violet

For SA-β-Galactosidase assay, cells were washed with PBS 1X, fixed for 5 min in 0.5% glutaraldehyde, rinsed twice in PBS 1X, and incubated at 37°C overnight in SA-β-Galactosidase staining solution as previously described [29]. For crystal violet staining, cells were washed with PBS 1X, fixed for 15 min in 3.7% formaldehyde and stained with crystal violet.

### Cell counts

After treatment, cells were fixed with 3.7% paraformaldehyde and stained with Hoechst (Sigma-Aldrich, USA). Images were automatically acquired by Operetta CLS High Content Analysis System (PerkinElmer, USA). The number of nuclei per well were counted using Columbus Image Data Storage and Analysis System (PerkinElmer).

### Animals

Eight weeks old C57B/6 males (Janvier Labs, France) were used. Intratracheal injection of Elastase 10 UI (MedChemExpress, USA) or NaCl 0.9% vehicle was performed, followed by intra-peritoneal injection of 2.5 mg/kg Benidipine (MedChemExpress) or vehicle (5% DMSO, 40% PEG 300, 5% Tween 80 and 50% NaCl 0,9%) every day from Monday to Friday. Three weeks after elastase injection, mice were necropsied. Lungs were inflated at constant pressure and fixed with 4% formaldehyde. At this step, lungs were excluded, if not properly inflated, for the mean linear intercept (MLI) analysis. They were then paraffin-embedded and slides were prepared as described in [30]. The mean linear intercept was assessed using HE stained slides as previously reported [13] at 3 different depths and MLI per mouse calculated. Mice were maintained in laminar-flow boxes under standard conditions (standard diet and water *ad libitum*) in our specific pathogen-free facility. Experiments were performed according to animal care guidelines of European and French laws. Protocols were authorized by the local animal ethics evaluation committee (CLB-2019-008) and by the French Ministry of Education and Research (Apafis#21449).

### Transcriptome analysis

Gene expression profiling has been performed by Microarrays using Whole Human Genome Microarrays 4 × 44K v2 (Agilent Technologies, USA) and the Agilent workflow for one-color gene expression. Briefly, 100 ng of total RNA, extracted from MRC5 cells with RNA NucleoSpin^®^ kit (Macherey-Nagel, France), were labelled with Cy3 dye using one-color Low Input Quick Amp Labeling Kit, (Agilent Technologies). After quality control validation, 1650 ng of Cy3-labeled cRNAs purified with RNeasy columns (Qiagen, USA) were hybridized on the 4 × 44K arrays for 17 hr at 65°C. Microarrays were washed and scanned with Agilent DNA microarray scanner G2565CA (Agilent Technologies). Fluorescence signals were quantified with Feature Extraction Software Version 10.5.1.1 (Agilent Technologies). Then Genespring GX 12.6 software (Agilent Technologies) was employed for data processing and data mining. Data normalization was done applying 75th percentile method. Microarray probes were filtered using Agilent flag filter to remove probes with raw signal below 10 in all the conditions tested. Transcriptomic analysis was performed on 3 independent replicates and differentially expressed genes were selected with fold change cutoffs > or < 1.5 and *t*-test *p* value < 0.05. Pre-ranked Gene Set Enrichment Analysis (GSEA) was performed on ranked list of fold change expression using GSEA v2.0.13 software using default parameters. All gene set files for this analysis were obtained from GSEA website (http://www.broadinstitute.org/gsea/).

### Statistical analysis

All statistical analyses and graphs were created with GraphPad Prism 9.3.1. Statistical analyses are indicated for each graph. Abbreviation: ns: non-significant; ^*^p < 0.05; ^**^p < 0.01; ^***^p < 0.001.

### Data availability statement

The data that support the findings of this study are available from the corresponding author upon reasonable request.

## Supplementary Materials

Supplementary Figures

Supplementary Tables
