# Supplementary material for: Benidipine calcium channel blocker promotes the death of cigarette smoke-induced senescent cells and improves lung emphysema
Source: Aging (Albany NY). 2023 Dec 12;15(23):13581–92. doi: 10.18632/aging.205259 (PMC10756105; doi:10.18632/aging.205259)
Supplement: Supplementary Figures [file aging-15-205259-s001.pdf]

SUPPLEMENTARY FIGURES

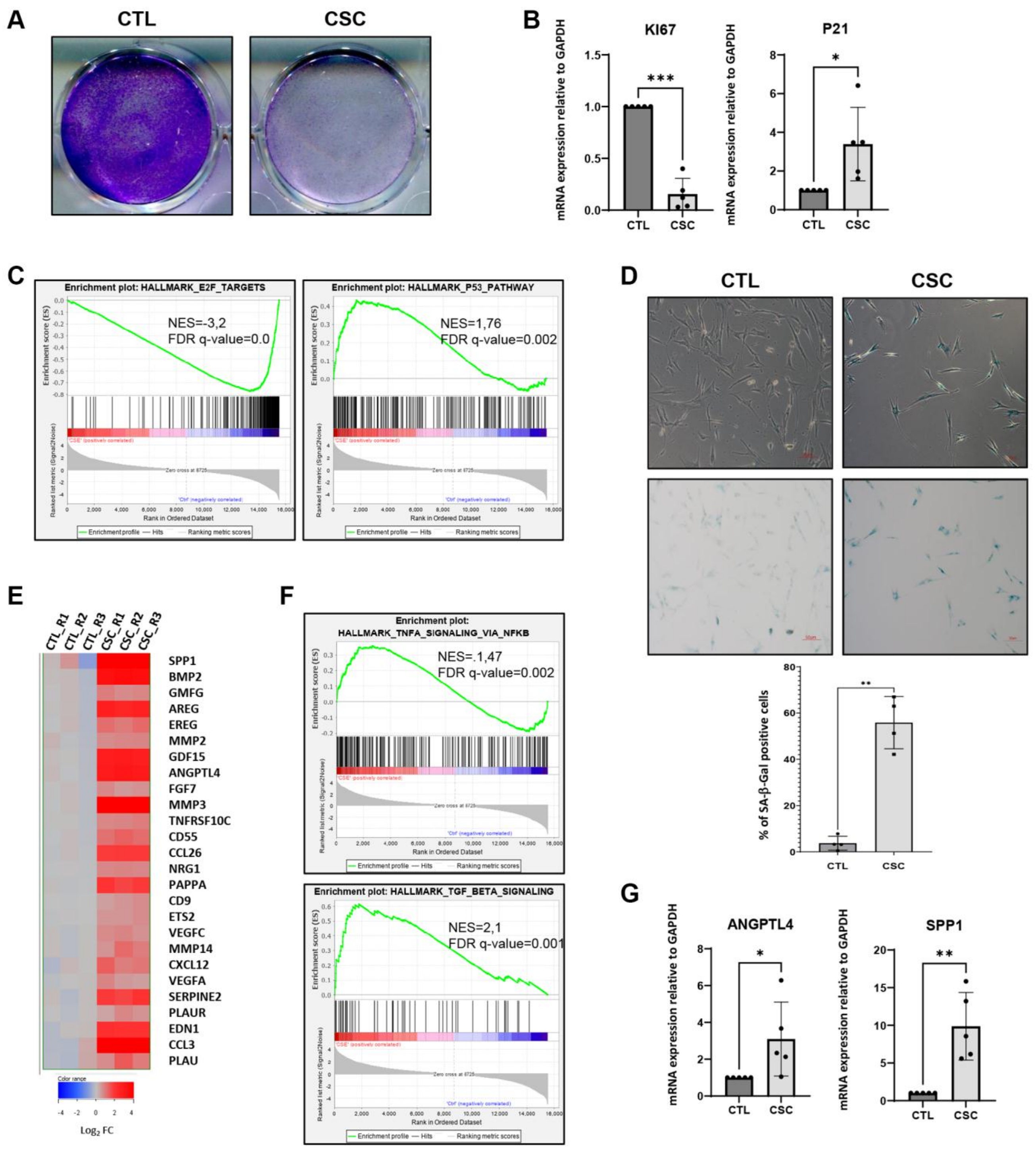

Supplementary Figure 1. Cigarette smoke condensates induce premature senescence. MRC5 human fibroblasts were treated 6 days with cigarette smoke condensates (CSC) at 40 µg/ml. Two to 4 days after the last treatment, various assays were performed: (**A**) Cells were PFA-fixed and crystal violet stained to visualize cell density. (**B**) RTqCR analysis against proliferation marker (KI67) and cyclin dependent kinase inhibitor p21 were performed on CSC-treated cells (CSC) and DMSO treated control cells (CTL). Results were normalized against GAPDH reference (mean ± SEM, paired *t*-test, *n* = 5). (**C**) Transcriptomic analysis were performed on CSC treated cells (CSC) and DMSO-treated control cells (CTL). GSEA enrichment plots from transcriptomic analysis of CSC- treated cells versus control cells are shown. Enriched pathway indicating a proliferation arrest are displayed. Name of the pathway are indicated on the top of plots. Normalized enrichment score (NES) and statistic *q*-values are indicated. (**D**) SA-β-Gal assays were performed on CTL and CSC-treated cells. Representative images are shown, as well as the percentage of positive cells. (mean ± SEM, paired *t*-test, *n* = 4). (**E**) Heatmap shows up-

regulation of numerous senescence SASP markers defined par MAYO clinic signature in CSC condition compared to CTL condition. R1 R2 R3 indicates 3 independent replicates. (**F**) GSEA enrichment plots of pathways linked to the SASP. (**G**) RTqCR analysis against SASP factors. Results were normalized against GAPDH (mean ± SEM, paired *t*-test, $n = 5$). $^{*}p < 0.05$; $^{**}p < 0.01$; $^{***}p < 0.001$.

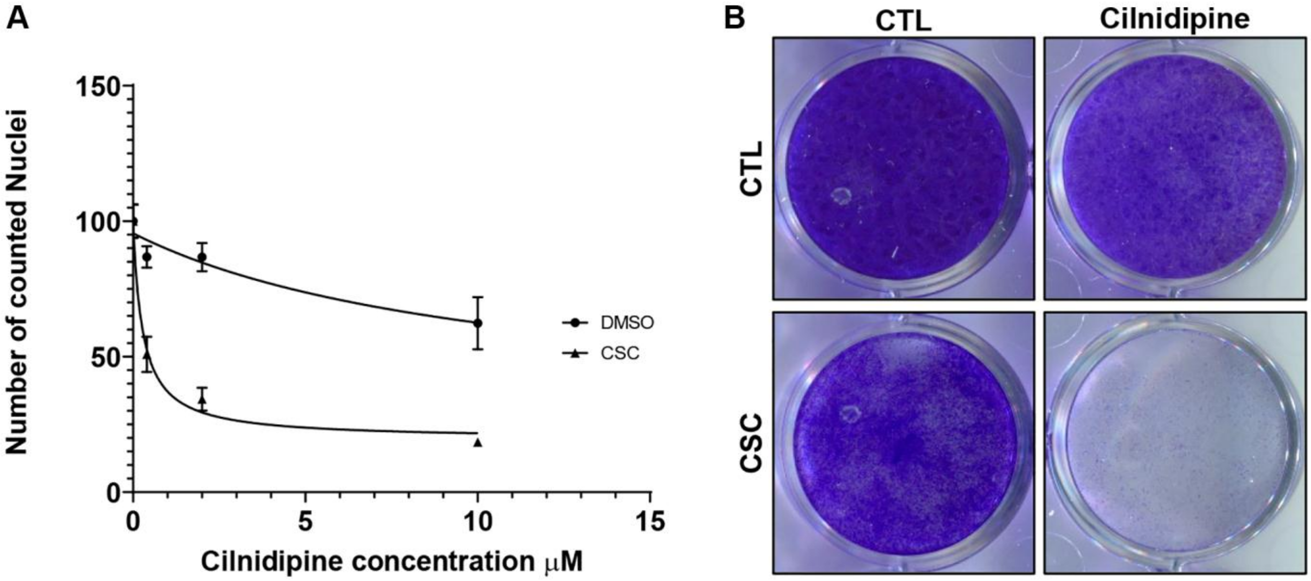

Supplementary Figure 2. Cilnidipine induces the death of CS-induced senescent cells. (**A**) Dose response curve of CaV blocker Cilnidipine senolytic activity. X axis shows Cilnidipine contractions used, Y axis shows percentage of cell count relative to control DMSO treated cells (% of counted nuclei) (mean ± SEM). (**B**) After indicated treatment, cells were fixed by paraformaldehyde and stained using crystal violet to visualize cell density.
