# Supplementary material for: Benidipine calcium channel blocker promotes the death of cigarette smoke-induced senescent cells and improves lung emphysema
Source: Aging (Albany NY). 2023 Dec 12;15(23):13581–92. doi: 10.18632/aging.205259 (PMC10756105; doi:10.18632/aging.205259)
Supplement: Supplementary Tables [file aging-15-205259-s002.pdf]

SUPPLEMENTARY TABLES

Supplementary Table 1. List of 134 molecules selected after the first round of screening.

2-Methoxyestradiol (2-MeOE2)	Cinacalcet	Erlotinib Hydrochloride	MLN2238	Raltitrexed
ABT-263 (Navitoclax)	Cinacalcet HCl	Ethacridine lactate monohydrate	MLN8237 (Alisertib)	Regorafenib
Afatinib (BIBW2992)	Cobicistat (GS-9350)	Felodipine	Mocetinostat (MGCD0103, MG0103)	Regorafenib hydrochloride
Afatinib dimaleate	CUDC-101	Fenbendazole	Motolimod (VTX-2337)	RG7388
Amlodipine	Curcumin	Fingolimod (FTY720)	Mycophenolate Mofetil	Rocilinostat (ACY-1215)
Amlodipine Besylate	Cyclosporin A	Flunarizine 2HCl	Nifedipine	Romidepsin (FK228, depsipeptide)
Anidulafungin	Cyclosporine	Fluvastatin	Nintedanib (BIBF 1120)	Rupatadine Fumarate
Aprotinin	Dabigatran etexilate mesylate	Fluvastatin Sodium	Nisoldipine	Salmeterol xinafoate
Azelnidipine	Dasatinib (BMS-354825)	Foretinib (GSK1363089)	Novobiocin Sodium	Saracatinib (AZD0530)
Azithromycin	Daunorubicin HCl	Ganetespib (STA-9090)	Olaparib (AZD2281, Ku-0059436)	Sertaconazole nitrate
Azithromycin Dihydrate	Dehydroepiandrosterone (DHEA)	Hydroxychloroquine Sulfate	Oltipraz	Suprofen
Bardoxolone methyl	Desloratadine	Ibuprofen Lysine	Orlistat	Telaprevir (VX-950)
Belinostat (PXD101)	Digoxin	Idarubicin HCl	Oxytocin	Tetracaine HCl
Benidipine HCl	Disulfiram	Idebenone	Pacritinib	TG101348 (SAR302503)
Benzydamine HCl	DL-α-Difluoromethylornithine (hydrochloride hydrate)	Imatinib (STI571)	Pamidronate Disodium	Tofacitinib (CP-690550, Tasocitinib)
Birinapant (TL32711)	Docetaxel Trihydrate	Imatinib Mesylate (STI571)	Panobinostat (LBH589)	Trandolapril
Cabozantinib (XL184, BMS-907351)	Domiphen Bromide	Ispinesib (SB-715992)	Pazopanib (GW-786034)	Trifluoperazine 2HCl
Cabozantinib malate (XL184)	Doxorubicin	Isradipine (Dynacirc)	PCI-24781 (CRA-024781)	Vandetanib (ZD6474)
Calcitriol	Drospirenone	Ivermectin	PCI-32765 (Ibrutinib)	Vemurafenib (PLX4032, RG7204)
Carfilzomib (PR-171)	Dyclonine HCl	K-115	Pelitinib (EKB-569)	Verapamil HCl
Carmofur	Ebastine	Lacidipine	Pimasertib (AS-703026)	Vinorelbine
CEP-18770	EMD-1214063	Lomitapide	Ponatinib (AP24534)	Vorinostat (SAHA, MK0683)
Cepharanthine	Entinostat (MS-275, SNDX-275)	Lovastatin	Pracinostat (SB939)	VX-680 (MK-0457, Tozasertib)
Cetrimonium Bromide (CTAB)	Entrectinib	LY2784544	Proflavine Hemisulfate	YM155
Cetylpyridinium Chloride	Enzastaurin (LY317615)	Mefloquine hydrochloride	Quetiapine Fumarate	Zinc Pyrithione
Chlorquinaldol	Epirubicin HCl	Mevastatin	R788 disodium	Zoledronic Acid
Cilnidipine	Erlotinib	Mitoxantrone HCl	Raloxifene HCl	

CS-induced lung senescent cells were treated with 10 µM of each 1,379 molecule contained in the FDA-approved library and the number of nuclei were automatically counted. We selected molecules that resulted in an approximate 40% decrease in stained nuclei and we excluded molecules known to have broad cytotoxic activity independently of senescence.

Supplementary Table 2. List of primers used.

Gene	Primer forward	Primer reverse	UPL#
GAPDH	AGCCACATCGCTCAGACAC	GCCCAATACGACCAAATCC	60
CDKN1A/p21	TCACTGTCTTGTACCCTTGTGC	GGCGTTTGGAGTGGTAGAAAT	32
SPP1	GAGGGCTTGGTTGTCAGC	CAATTCTCATGGTAGTGAGTTTTCC	18
ANGPTL4	GACAAGAACTGCGCCAAGA	GCCGTTGAGGTTGGAATG	49
mCDKN1A/p21	TGCGCTTGGAGTGATAGAAA	AACATCTCAGGGCCGAAA	NA
mGAPDH	CCTGCTTCACCACCTTCTTG	TGTCCGTCGTGGATCTGAC	NA
